# Vitamin D status, supplementation, and multiple sclerosis: a systematic review and meta-analysis

**DOI:** 10.3389/fimmu.2026.1775270

**Published:** 2026-03-30

**Authors:** Yanyan Li, Ying Xu, Xiaorui Pei, Ziqi Liu, Dan Gao, Lili Ma, Yan Wang, Yanan Fang, Ying Cai

**Affiliations:** 1 Department of Neurology Ward, Chaoyang Central Hospital, Chaoyang, Liaoning, China; 2 Clinical Medicine Major, Xinhua Clinical College, Dalian University, Dalian, Liaoning, China; 3 Department of Cardiology, Chaoyang Central Hospital, Chaoyang, Liaoning, China

**Keywords:** expanded disability status scale, MS, multiple sclerosis, supplementation, vitamin D, VDRpolymorphisms

## Abstract

**Background:**

Multiple sclerosis (MS) is a chronic immune-mediated neurodegenerative disorder of the central nervous system and a leading cause of disability among young adults. The disease exhibits considerable heterogeneity in clinical progression, with some patients experiencing more aggressive disease activity and a rapid decline in quality of life. Vitamin D plays a key role in immune regulation, and evidence suggests that its deficiency constitutes a significant environmental risk factor for immune-mediated conditions such as MS. While there are controversies regarding the role of serum 25(OH)D in MS as well as vitamin D3 supplements in controlling relapse and disability improvement during treatment. Therefore, our current meta-analysis aims to examine the relationship between serum 25(OH)D levels—and their modulation through supplementation—and multiple sclerosis.

**Methods:**

Comprehensive literature review was performed from conception to November 17, 2025, employing various online databases including PubMed, Cochrane Library, Web of Science, and EMBASE. The relevant studies about MS and serum 25-hydroxyvitamin D (25(OH)D) level or vitamin D3 supplementation. This meta-analysis incorporated 40 research examining the correlation between serum 25(OH)D levels and multiple sclerosis, as well as 22 studies investigating the effects of vitamin D3 supplementation on multiple sclerosis.

**Results:**

1) The 25(OH)D levels in patients with MS were significantly lower than those in healthy controls. In the analysis of disease subtypes, patients with relapsing-remitting MS (RRMS) had significantly higher 25(OH)D levels than those with secondary progressive MS (SPMS), but no significant difference was observed compared to patients with primary progressive MS (PPMS). Additionally, RRMS patients had significantly lower 25(OH)D levels during relapse than during remission. No significant seasonal fluctuation in 25(OH)D levels was observed in MS patients; 2) Multivariable-adjusted analysis comparing the highest versus lowest serum 25(OH)D categories revealed that higher serum 25(OH)D levels were associated with a lower risk of MS onset and lower disability scores (EDSS), but no significant association was found with disease activity. 3) In the intervention analysis, overall, vitamin D3 supplementation did not significantly reduce the annualized relapse rate (ARR) or improve EDSS scores. However, subgroup analysis indicated that high-dose vitamin D3 supplementation significantly reduced the ARR, whereas low-dose supplementation showed no such effect. Multivariable-adjusted analysis further confirmed that vitamin D3 supplementation was significantly associated with a reduced risk of MS relapse, and this benefit was similarly observed only in the high-dose supplementation group.

**Conclusions:**

This systematic analysis confirms that patients with MS exhibit significantly lower serum 25(OH)D levels compared to healthy controls, with notable reductions observed during clinical relapses. A clear gradient exists across disease subtypes, with RRMS patients showing higher levels than those with SPMS. An inverse correlation was demonstrated between serum 25(OH)D levels and both the risk and severity of MS. Critically, while high-dose vitamin D3 supplementation is associated with a reduced ARR and overall relapse risk, neither supplementation compared to placebo nor dosage comparison significantly affected relapse rates during the study period or final disability scores. These findings suggest a complex, dose-dependent role of vitamin D in modifying relapse risk without a clear impact on short-term disability progression in established MS.

**Systematic Review Registration:**

https://www.crd.york.ac.uk/PROSPERO/login, identifier CRD420251273119.

## Introduction

MS is a chronic inflammatory, autoimmune demyelinating disorder of the brain and spinal cord, characterized by multifocal lymphocytic infiltration leading to myelin destruction, along with damage to oligodend rocytes, axons, and neurons. Pathological changes are predominantly observed in white matter, although gray matter infiltration can also occasionally be seen (1–3). These pathological processes can result in significant impairment of motor, sensory, autonomic, and cognitive functions, making MS one of the leading causes of disability in young adults.

Common neurological manifestations include sensory abnormalities, optic neuritis, visual impairment, limb weakness, ataxia, bladder dysfunction, cognitive impairment, and fatigue (4). The clinical course of MS is highly heterogeneous, with some patients experiencing high disease activity that leads to a rapid decline in quality of life (5). Similar to other autoimmune diseases, the etiology of multiple sclerosis remains incompletely understood.

It is generally believed to be an autoimmune disorder that occurs in genetically susceptible individuals following exposure to specific environmental factors (6). Studies have confirmed that both genetic and environmental factors contribute to the development of the disease (7, 8). Polymorphisms in the vitamin D receptor (VDR) gene have been extensively studied as important genetic factors that modulate individual responsiveness to vitamin D and influence susceptibility to MS. The VDR is the key molecule mediating the biological effects of vitamin D. Common single nucleotide polymorphisms (e.g., FokI, BsmI, ApaI, TaqI) in its encoding gene may alter the receptor’s transcriptional activity, protein stability, or ligand-binding efficiency, thereby influencing the immunomodulatory and neuroprotective functions of vitamin D (9, 10). These genetic variations may lead to differential biological responses to similar vitamin D levels among individuals, partly explaining the inconsistent findings in observational studies and supplementation trials (11, 12). Therefore, assessing VDR polymorphisms contributes to a more comprehensive understanding of the role of vitamin D in the pathogenesis of MS and may inform the development of personalized intervention strategies based on genetic background in the future. Since genetic factors are generally unmodifiable, identifying environmental factors that influence the onset and progression of multiple sclerosis and actively intervening in these factors are crucial for understanding the origin of the disease and improving patient prognosis. 25(OH)D is a key regulator of calcium and phosphorus homeostasis, essential for maintaining bone health.

Experimental evidence also supports its role in immunomodulation, cellular differentiation, and neural development (13–15). Through vitamin D receptors expressed on activated T lymphocytes, the active form of vitamin D can suppress T helper type 1 (Th1) cell activity—a pathway implicated in the pathogenesis of multiple sclerosis (MS), where excessive Th1 responses are believed to drive disease progression (16–18). Thus, vitamin D functions as an important immunoregulator, and its deficiency has been recognized as a significant environmental risk factor in MS.

Although there is substantial evidence regarding the role of 25(OH)D in MS, current conclusions remain inconsistent. For example, a study by Gao et al. indicated that serum 25(OH)D levels in MS patients were significantly lower than in healthy controls (19), while research by Naiini et al. suggested higher 25(OH)D levels in the MS group compared to the control group (20). Several prospective studies have shown that lower serum 25(OH)D levels prior to the onset of MS may increase the risk of developing the disease (21–23), although other studies have failed to confirm any neuroprotective effect of 25(OH)D in MS (24, 25). The impact of 25(OH)D on disease activity and severity indicators in MS, such as relapse rate, remains controversial. Some studies suggest that low 25(OH)D levels may serve as a biomarker for increased disease activity, aiding in prognosis assessment and treatment decisions in early MS (26–28). Additionally, vitamin D3 supplementation may produce several beneficial immunomodulatory effects, including promoting regulatory T cells (Tregs) (29) and the anti-inflammatory cytokine IL-10 (30, 31), reducing pro-inflammatory Th17 cells (32) and IL-17 (33), and lowering B-cell immunoreactivity (34). However, these findings have yet to reach a consensus. At the clinical level, some studies have shown that vitamin D3 supplementation can reduce the risk of MS relapse, but its relationship with the prediction of disability progression remains unclear (35, 36). This issue is particularly significant for patients with chronic progressive MS, who often exhibit the lowest 25(OH)D levels (37). Cross-sectional studies suggest a negative correlation between serum 25(OH)D levels and disability, though the causal relationship remains uncertain (38, 39). In relapsing-remitting MS, greater sun exposure—the primary source of vitamin D— is associated with a reduced risk of progression on the EDSS, while in progressive MS, it is linked to a lower risk of progression on the Patient -Determined Disease Steps (PDDS) scale (40, 41). However, no association has been observed between serum25(OH)D levels and the MS Severity Scale (MSSS) or EDSS in African Americans (42). Moreover, a prospective study conducted in Tasmania found that after adjusting for baseline EDSS, the negative correlation between recent EDSS progression and serum 25(OH)D levels was no longer maintained (43). Therefore, clarifying the influence of serum 25(OH)D levels on the onset, disease activity, and disability progression in MS is crucial, as it serves as a prerequisite for promoting effective lifestyle interventions and supplementation strategies.

A substantial body of evidence has established that vitamin D deficiency is prevalent among patients with MS and is linked to an increased risk of disease onset and progression, possibly through its immunomodulatory and neuroprotective properties. However, several critical gaps persist. First, the relationship between serum 25(OH)D levels and MS appears to vary across clinical subtypes (e.g., relapsing- remitting vs. progressive forms), but systematic comparisons are limited and results are inconsistent. Second, while some studies suggest that vitamin D3 supplementation may reduce relapse rates, the optimal dose, duration, and target population remain poorly defined. Inconsistent findings regarding its effect on disability progression, as measured by the EDSS, further complicate clinical translation. Third, previous meta-analyses have often pooled heterogeneous study designs without stratified analyses by disease subtype, disease activity, or supplementation dosage, limiting the ability to draw nuanced conclusions. Therefore, despite numerous publications, there is a lack of a comprehensive synthesis that simultaneously examines 25(OH) D status across MS subtypes and evaluates the dose-dependent efficacy of vitamin D3 supplementation on both relapses and disability outcomes.

This meta-analysis addresses these gaps by integrating evidence from both observational and interventional studies with pre-specified subgroup analyses. Unlike previous reviews, we explicitly compare serum 25(OH)D levels across RRMS, SPMS, and PPMS subgroups, evaluate differences between relapse and remission phases, and assess the impact of vitamin D3 supplementation stratified by dose (high vs. low) on clinical endpoints including ARR and EDSS. Our study thus provides a more granular understanding of the role of vitamin D in MS, VDR gene and MS, clarifying for whom and under what conditions vitamin D monitoring or supplementation may be most beneficial.

## Materials

### Search strategy and study selection

A systematic literature search was conducted from database inception through November17, 2025, utilizing the following electronic databases: PubMed, the Cochrane Library, Web of Science, and EMBASE. Additional records were identified by reviewing the reference lists of included studies and relevant articles not captured by the primary search. The search strategy combined terms for multiple sclerosis (“Multiple sclerosis” or “MS”) with terms related to vitamin D (“vitamin D”, “25(OH) vitamin D”, “25-hydroxylvitamin D”, “vitamin D3 supplementation”,”VDR”, “VDR polymorphisms”) using Boolean operators (“AND”, “OR”).In this manuscript, we use specific terminology to distinguish between vitamin D status and supplementation. “25(OH)D” refers to serum 25-hydroxyvitamin D, the standard biomarker for assessing circulating vitamin D levels. “Vitamin D3” or “cholecalciferol” refers to the specific form of vitamin D used in supplementation interventions. The term “vitamin D” is used only in general contexts (e.g., “vitamin D metabolism”) or when referring to the broader field of study. Throughout the manuscript, we maintain this distinction to ensure clarity between observational measures of vitamin D status and interventional vitamin D3 supplementation.

**Figure 1:**
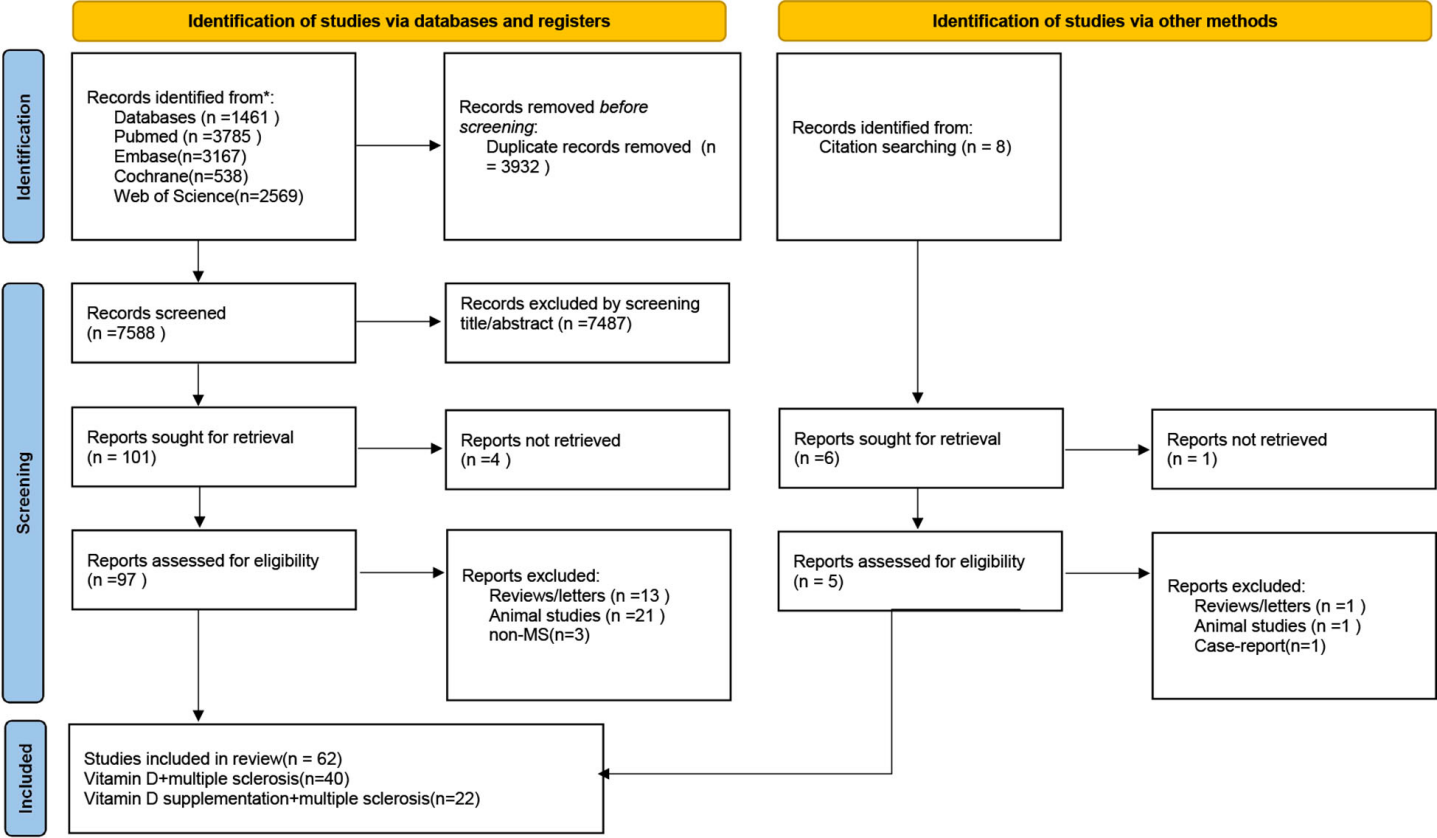
Preferred Reporting Item for Systematic Reviews and Meta-Analysis (PRISMA) guideline.

Two investigators independently screened titles and abstracts retrieved from the search. Publications meeting preliminary criteria underwent full-text review against predefined eligibility criteria. The study wasperformed in accordance with the Preferred Reporting Items for Systematic Reviews and Meta-Analyses (PRISMA) guidelines (44) (**Figure 1**). The study was preregistered in PROSPERO (see the online supplemental file-PROSPERO, CRD420251273119).

### Protocol registration

This systematic review was registered with PROSPERO (CRD420251273119) on December 25, 2025. The literature search was initiated in September 2025 and completed on November 17, 2025, prior to the completion of data extraction. According to PROSPERO guidance, registration is considered prospective as long as it occurs before the completion of data extraction. At the time of registration (December 25, 2025), data extraction was still ongoing, and no synthesis of findings had been performed. The registered protocol specified a search date of “before November 17, 2025,” which aligns with the actual conduct of the search. The study end date (January 28, 2026) in the PROSPERO record refers to the anticipated completion of data analysis and manuscript preparation, which occurred after manuscript submission. No deviations from the registered protocol occurred during the conduct of this review.

## Methods

### Data extraction and study quality

#### Inclusion criteria

The inclusion criteria were as follows: (1) studies published in English with publicly accessible data; (2) case-control or prospective cohort studies; (3) investigation of the association between serum 25(OH) D (or vitamin D3 supplementation) and multiple sclerosis (MS); and (4) provision of effect estimates, including weighted mean difference (WMD), odds ratio (OR), or relative risk (RR), with corresponding 95% confidence intervals (CIs).

The diagnosis of MS was primarily based on the 2017 McDonald Criteria (45). Key outcome measures included the EDSS and the ARR. Disease course was categorized as RRMS, SPMS, or PPMS based on standard clinical definitions emphasizing relapse activity and progression of neurological disability.

#### Exclusion criteria

Two reviewers independently retrieved data from pertinent articles utilizing data extraction forms that encompassed details such as authorship, publication year, research design, sample size, various biomarker levels, and patient outcomes. We excluded: (1) duplicate or irrelevant papers; (2) reviews, letters, or case reports; (3) non-original research (editorials, reviews, or commentaries); and (4) studies involving non-human subjects. The summary of the patient, intervention/exposure, comparator, outcome, and study design (PICOS) criteria for inclusion and exclusion of studies to a systematic review are presented in **Table 1**.

**Table 1:** The summary of the patient, intervention/exposure, comparator, outcome, and study design (PICOS) criteria for inclusion and exclusion of studies in a systematic review.

PICOS parameter	Inclusion criteria	Exclusion criteria
Population	Target Patient Population:Individuals diagnosed with Multiple Sclerosis (MS), encompassing all clinical subtypes (Relapsing-Remitting MS [RRMS], Secondary Progressive MS [SPMS], Primary Progressive MS [PPMS]) and disease phases (clinical relapse, remission). Control Population:Age- and sex-matched healthy adults. Prespecified Subgroup Analyses:Subgroup analyses will be conducted based on factors including MS subtype, baseline disease activity, baseline vitamin D status, and geographic latitude.	Children and adolescents<16 years old, pregnant women with multiple sclerosis, individuals with any intellectual disabilities, any eating disorders, or any other neurological disorders
Intervention/exposure	Observational Study Component (Exposure): Serum or plasma concentration of circulating 25-hydroxyvitamin D [25(OH)D], used as a biomarker of vitamin D nutritional status. Interventional Study Component (Intervention): Oral vitamin D supplementation (including cholecalciferol [D_3_] and ergocalciferol [D_2_]). The analysis will specifically focus on the effects of different dosing regimens, with explicit definition and comparison of "high-dose" versus "low-dose/standard-dose" supplementation.	Vitamin D applied within multiple nutrients supplementation
Comparison	Observational Study Component:Comparison with a healthy control group; or comparisons within the MS cohort between different disease subtypes or disease activity states (e.g., active vs. inactive phase). Interventional Study Component: Placebo control. Usual care or standard of care control. Direct comparison between different vitamin D supplementation dosing regimens (e.g., high-dose vs. standard-dose).	Lack of comparison
Outcome	Association Outcome: Risk of developing MS (reported as Odds Ratio [OR], Relative Risk [RR], or Hazard Ratio [HR]). Disease Activity Outcome: Annualized Relapse Rate. Disability Progression Outcome: Change in the Expanded Disability Status Scale (EDSS) score over time.	Studies without relevant outcome measures or with only unrelated outcome measures.
Study design	Interventional Studies: Randomized Controlled Trials (RCTs) evaluating the effect of vitamin D supplementation. Observational Studies: Cohort studies (prospective or retrospective) and case-control studies that investigate the association between serum 25(OH)D levels and MS risk or disease course.	Case reports, case series, narrative reviews, systematic reviews/meta-analyses (although their reference lists will be screened for eligible studies), conference abstracts, commentaries, letters, non-human (animal or *in vitro*) studies, and studies not published in the English language.

#### Data extraction

The gathered data includes the lead author, publication year, area, age, gender, sample size, study methodology, impact estimates, and adjustments. Effect estimates adjusted for primary confounding variables, together with their 95% confidence intervals (CIs), were extracted for the comparison of the highest versus lowest categories of serum 25(OH)D levels. For studies reporting relative risks (RRs) per unit increase in 25(OH)D, these were converted to RRs for highest versus lowest categories where possible, using established methods. All RRs presented in this meta-analysis represent the risk associated with higher 25(OH)D levels compared to lower levels (highest vs. lowest category), unless otherwise specified. Furthermore, the serum 25(OH)D concentrations (standard deviation [SD]) were acquired for both multiple sclerosis patients and control subjects. In contrast, the unit of 25(OH)D was standardized to “ng/mL” across all trials. The investigation of vitamin D3 supplementation and multiple sclerosis examined the following components: we extracted data on vitamin D3 assessment methods, relative risks (RRs) or weighted mean differences (WMDs) for clinical outcomes (relapse rate, disability progression), and variables adjusted for in the analysis.

#### Quality assessment

Every author independently appraised the assessment of quality and risk of bias in the included studies utilizing the Newcastle-Ottawa Scale (NOS) (46). The framework encompasses three primary aspects: the selection of research cohorts, the comparability among various cohorts, and the identification of either the exposure or result of the study cohorts. All disagreements were resolved via dialogue. The scale ranges from 0 to 9 stars. Studies with scores of 0–4 were considered low-quality, 5–6 were considered moderate-quality, and 7–9 were considered high-quality. A low NOS score typically implies that the study is of poor quality.

### Statistical analysis

The statistical analysis was performed utilizing STATA 17.0 and Review Manager 5.3 for data synthesis and evaluation. For continuous data, mean ± standard deviation is displayed, along with the mean with weighted mean difference (WMD) and 95% confidence intervals (CIs). When the incorporated research presented data as medians and interquartile ranges, the mean and standard deviation were calculated following the methodology developed by Wan and colleagues (47). Statistical significance was defined a p- values<0.05, and 95% confidence intervals were supplied. The weighted mean difference (WMD) was used as the metameter and the standard deviation (SD) was considered in evaluating the precision and significance of that point of estimate. Whereas heterogeneity across studies was evaluated using Cochrane- based Q and I2 tests. Data followed by p < 0.05 or I2>50% were considered to denote statistically significant heterogeneity, and were subjected to a randomized-effects model. Otherwise, a fixed-effects model was used.A funnel plot was used to determine publication bias.

This meta-analysis included both observational studies (examining associations between serum 25(OH)D levels and MS outcomes) and interventional studies (examining effects of vitamin D3 supplementation). These two types of studies were analyzed separately to address distinct research questions: observational studies for associations between serum 25(OH) D status and MS risk/severity, and interventional studies for efficacy of supplementation on clinical outcomes. No quantitative synthesis combining observational and interventional data was performed. Findings from observational and interventional analyses are presented separately in the Results section and interpreted distinctly in the Discussion, given their inherent methodological differences and varying susceptibility to confounding.

## Results:

### Literature search and study characteristics

The initial search yielded 11,520 citations. After removing duplicates, 7,588 records remained. Screening of titles and abstracts resulted in 101 reports sought for retrieval, of which 97 were assessed for eligibility in full text. Following exclusions (13 reviews, 21 non-human studies, and 3 studies not related to MS), 62 articles met the eligibility criteria and were included in the qualitative synthesis (**Figure 1**).

A meta-analysis was subsequently performed on 40 studies examining serum 25(OH)D levels and MS, and 22 studies investigating vitamin D3 supplementation and MS relapse. In the 22 eligible studies on vitamin D3 supplementation in multiple sclerosis, patients in the control group received either a low dose of vitamin D3 supplementation (range: 400–1000 IU per day) or placebo. Patients in the treatment group were administered a high dose of vitamin D3 supplementation (range: 2857–20,400 IU per day). Due to substantial heterogeneity in the definitions of vitamin D3 dosing across the included studies, the classifications of “ high dose” and “ low dose” in this meta-analysis were based on the grouping criteria used in each original trial, without applying a pre-specified uniform dose threshold. Detailed dose ranges for each study are provided in **Table 2**. Vitamin D3 treatment duration ranged from 24 to 192 weeks. The observation period in this study corresponds to the follow-up duration reported in each original trial. In subgroup analyses, no uniform cut-off for follow-up time was applied; all analyses were based on outcomes reported at the final follow-up in each included study. The specific follow-up duration for each study is shown in **Table 2**. Characteristics of the included studies are summarized in **Tables 3**, **2**. Sun exposure is a major determinant of serum 25(OH)D levels and thus a potential confounder in the relationship between serum 25(OH)D levels and MS. While we extracted available data on study location and season of sampling where reported, individual-level data on sun exposure behavior, UV dose, or time outdoors were not consistently available across studies. Therefore, we could not quantitatively adjust for this variable in the pooled analysis. Study quality scores ranged from 6 to 9, indicating that all included studies were of moderate to high quality. .

**Table 2:** Vitamin D3 supplementation and MS.

Study and year	Country	N total	Intervention	Other treatments	Duration	Vitamin D group/ High dosage	Placebo group/ Low dosage	Outcome measure
Thouvenot E 2025	France	271	Patients were randomized to receive oral cholecalciferol and placebo	none	24 months	Oral cholecalciferol 100000IU every 2 weeks	placebo	Disease activity,defined as occurrence of a relapse and/or MRI activity
Munger KL 2004	USA	346	Patients were randomized to a low-dose group and a high-dose group	none	48 months	>400IU/day	<400IU/day	MS relapses
Sandra D 2023	USA	172	Patients were randomized to receive low dose vitamin D3 (LDVD) or high dose vitamin D3 (HDVD)	none	96 weeks	5000IU/day (HDVD)	600IU/day (LDVD)	The primary outcome was the proportion that experienced a confirmed relapse and analyses used Kaplan Meier and Cox proportional hazards models.
Hupperts R 2019	Germany	229	Patients were randomized to receive vitamin D supplementation and placebo	IFN-β-1a	48 weeks	14,007 IU/d vitamin D3 + SC IFN-β;	placebo+SC IFN-β	EDSS, number of new GE lesions,relapses
Derakhshandi H 2012	Iran	60	Patients were randomized to receive vitamin D3 and placebo	none	12 months	50,000IU of vitaminD3 weekly	received a placebo weekly	Number of Relapses
Miele G 2022	Italy	136	According to vitamin D intake, we divided our population into two groups: users and non-users	crelizumab	12 months	Not defined	Without vitamin D supplementation	disease activity and relapse
Stein MS 2011	Australia	23	Patients were randomized to a low-dose group and a high-dose group	none	24 months	High-dose vitamin D (6,000 IU) daily	low-dose (1,000 IU) vitamin D daily	cumulative number of new GE lesions and change in the total volume of T2 lesions.change in EDSS and the number of relapses
DṎrr J 2019	Germany	41	Patients were randomized to a low-dose group and a high-dose group	IFN-β-1a	18 months	high- (20,400IU) cholecalciferol supplementation	low dose (400 IU) cholecalciferol supplementation	disease activity
Achiron 2014	Israel	158	Patients were randomized to receive alfacalcidol and placebo	none	6 months	Alfacalcidol (1 mcg/d)	Placebo	neurological examination was scored by the EDSS. fatigue was evaluated by the FIS
Shaygannejad V 2012	Iran	50	Patients were randomized to receive vitamin D supplementation and placebo	disease modifying therapy	12months	escalating calcitriol doses up to 0.5µg/day	placebo	effect on the EDSS score or relapse rate
Soilu-Hanninen 2020	Finland	32	Patients were randomized to receive vitamin D supplementation and placebo	IFN-β-1a	52 weeks	20,000 IU of cholecalciferol	placebo	disease activity
Kampman MT 2012	Norway.	71	Patients were randomized to receive vitamin D supplementation and placebo	Natalizumab glatiramer acetate IFN-β-1a	Weeks 48 and 96	20,000 IU vitamin D3 weekly	placebo	assessed the effect of the intervention on serum 25(OH) D, ARR, EDSS
Burton JM 2010	Canada	49	Patients were randomized to receive vitamin D supplementation and placebo	interferon, glatiramer acetate	52 weeks	patients received escalating vitamin D doses up to 40,000 IU/day over 28 weeks, followed by 10,000IU/day(12 weeks), and further downtitrated to 0 IU/day	placebo	immunologic biomarkers, relapse events, and EDSS score
Galus W 2023	Poland	133	Participants were divided into a case group and a control group based on whether they received vitamin D supplementation	Interferon-beta Glatirameracetate Dimethylfumarate Teriflunomide Fingolimod Natalizumab Ocrelizumab Alemtuzumab	24 months	cholecalciferol, which was taken orally,doses ranged from 1000 to 4000 IU daily	Without vitamin D supplementation	disability status expressed by EDSS, number of relapses and time to relapse
Røsjø E 2015	Norway	68	Patients were randomized to receive vitamin D supplementation and placebo	IFN-β-1a glatiramer acetate natalizumab	96 weeks	VitaminD3 supplementation(20,000 IU/ week)	placebo	inflammation marker,ARR,EDSS
B€acker-Koduah P 2020	Germany	67	Patients were randomized to a low-dose group and a high-dose group	none	18 months	high-dose (20 400 IU) vitamin D3 supplementa tion taken every other day	low-dose (400 IU) vitamin D3 supplementa tion taken every other day	inflammation marker, EDSS
Sotirchos 2015	USA	40	Patients were randomized to a low-dose group and a high-dose group	IFN-β-1a Glatiramer acetate Natalizumab Fingolimod Abatacept	6 months	10,400IU cholecalciferol daily	800IU cholecalciferol daily	relapses,inflammation marker
Gargari BN 2015	Iran	32	Before treatment and after treatment	none	2 months	Treatment group received an oral dose of 50,000IU of vitamin D every week .	After treatment	Change in EDSS
Amirinejad R 2021	Iran	31	Before treatment and after treatment	none	2 months	received an oral dose of 50,000 IU of vitamin D every week .	After treatment	Change in EDSS
Mosayebi G 2011	Iran	59	Patients were randomized to receive vitamin D supplementation and placebo	IFN-β-1a	6 months	received 300,000 IU vitamin D3 every month	placebo	EDSS and number of gadolinium-enhancing lesions
Kotb MA 2019	Egypt	35	Before and after treatment	none	12 months	Participants received 10,000 IU of cholecalciferol daily	After treatment	Change in EDSS score and Depressive symptoms
Camu M 2019	France	181	Patients were randomized to receive vitamin D supplementation and placebo	none	96 weeks	Patients received high-dose oral cholecalciferol 100,000 IU	placebo	ARR, MRI parameters, and change in EDSS

EDSS, Expanded Disability Status Scale score.

ARR, Annualized Relapse Rate.

GE, Gadolinium Enhancement.

**Table 3:** Characteristics of studies included in this meta-analysis.

Study and year	Country	Study type	N total	Case(ng/ml)	Control(ng/ml)	RR(MS/activity/ EDSS)	Quality-NOS
Vitamin D and MS							
Moghtaderi A.2013	Iran	case-control	110	10.64 ± 9.2	13.23 ± 17.56		9
AKTÜRK T 2019	Turkey	cross-sectional study	50	25.98+-9.64	33.2+-9.06		8
Tarasiuk J 2019	Poland	case-control	76	18.9+-6.96	19.47+-7.78		7
Pandit L 2013	India	case-control	218	39.01+-5.18	46.81+-4.89	0.28 (0.11,0.68)	9
Lucas RM 2011	Australia	case-control	611	75.1+-31.9	80.4+-31.4	0.93(0.86,1)	9
Pihl-Jensen G 2015	Denmark	cross-sectional	164	25.35+-16.37	19.1+-9.04		6
Skalli A 2017	Morocco	case-control	259	11.69+-6.97	12.98+-6.58		7
Gao ZD 2024	China	case-control	36	25.41+-7.46	34.78+-6.5		7
Naiini MR 2024	Iran	case-control	76	47.86±1.40-	44.19±1.21-		8
Khedr AMB 2022	Egypt	case-control	100	15.49+-6.19	40.16+-20.18		8
Akbay GD 2020	Turkey	case-control	112	8.87+-4.02-	18.86+-11.04-		8
I.A.F.van der Mei2007	Australia	case-control	408	51.4+-20.3	53.1+-21.1		8
Bettencourt A 2017	Portugal	case-control	442	15.96+-8.8	22.16+-9.36	0.97(0.96,0.98)	7
Yeh WZ 2024	Australia	case-control	66	88.2+-11.54-	70.79+-6.01		8
Correale J 2009	Argentina	case-control	88	47.3+-9.0	61.2+-5.6		8
Bhargava P 2017	USA	case-control	54	22.08+-7.88	22.36+-8.0		8
Jaalkhorol M 2024	Switzerland	case-control	62	22.63+-4.92	25.15+-3.1		7
Salzer J 2012	Sweden	case-control	576	16.6+-8.97	16.31+-10.74	0.39(0.16,0.98)	8
Niino M 2015	Japan	case-control	110	60.2+-16.8	71.2+-21.8		9
Agnello L 2018	Italy	case-control	245	21.8+-7.2	39.1+-9.3		7
Martinelli V 2014	Italy	retrospective study	100	53.79+-29.13	56.78+-30.03	2.04(0.96,4.36)	8
Derakhshandi H 2012	Iran	case-control	60	13.7+-6.24	16.42+-5.1		6
Kubicka K 2013	Poland	case-control	30	16.91+-8.44	25.28+-8.79		8
Vandebergh M 2022	Belgium	–	–	–	–	0.28(0.12,0.4)	
Ascherio A 2014	Germany	–	–	–	–	0.61(0.44,0.83)	
Vitamin D and disease activity							
Giordano A 2024	Italy					2.36(1.3,3.88)	
Miele G 2022	Italy					4.48(1.17,20.01)	
Fitzgerald KC 2015	USA					0.8(0.6,1.06)	
Muris AH 2016	The Netherlands					1.02(0.95,1.1)	
Ascherio A 2014	Germany					0.74(0.46,1.16)	
Ferre L 2018	Italy					0.21(0.01,2.59)	
Vitamin D and EDSS							
I.A.F. van der Mei2007	Australia					3.07(1.37,6.9)	
Thouvenot E 2014	France			–	–	2.5(1.04,5.88)	
Weinstock-Guttman B 2010	USA				–	3.72(1.07,12.9)	
Bettencourt A 2017	Portugal					0.8(0.69,0.92)	

### The combined WMD of serum 25(OH) D levels between multiple sclerosis patients and controls during relapse and remission phases of MS

**Figure 2:**
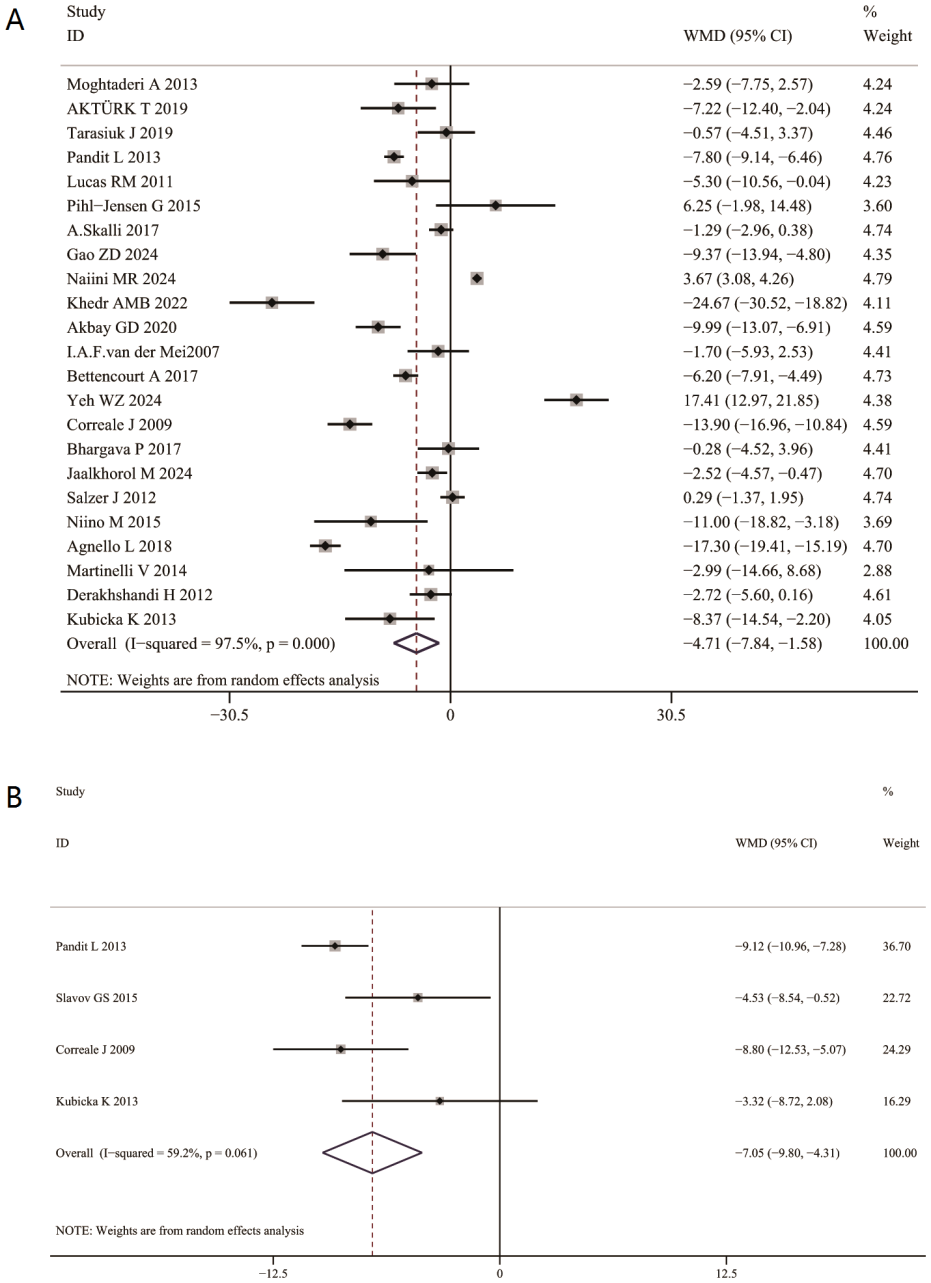
Association between serum 25(OH) D levels and multiple sclerosis (MS) patients. **(A)** serum 25(OH) D levels between multiple sclerosis (MS) patients and controls. **(B)** serum 25(OH) D levels between relapse and remission phases of MS.

The aggregated weighted mean difference (WMD) indicated that serum 25(OH) D levels were significantly lower in patients with MS compared to healthy controls(WMD=-4.54[-7.57,-1.51]) (**Figure 2A**). In subgroup analysis, serum 25(OH) D levels were markedly decreased in relapsing-remitting patients during relapse compared to remission (WMD = -7.05 [-9.8, -4.31]). (**Figure 2B**).

### The aggregated WMD of the serum 25(OH) D between RRMS group, PPMS group, SPMS group

**Figure 3:**
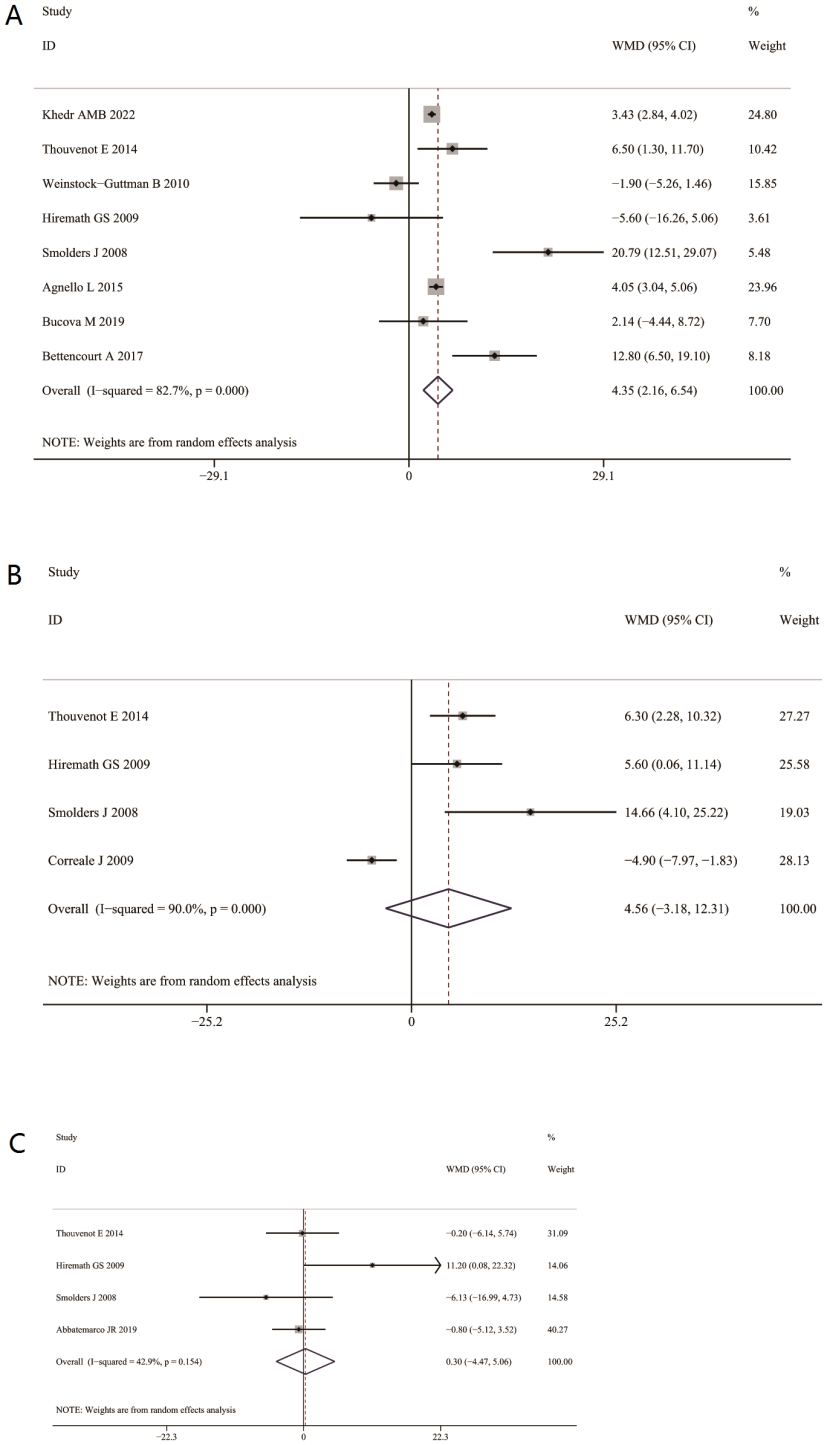
Association of 25(OH) D levels with subtypes of multiple sclerosis. **(A)** Association of serum 25(OH) D levels between relapsing-remitting multiple sclerosis (RRMS) and secondary progressive MS(SPMS). **(B)** Association of serum 25(OH)D levels between relapsing-remitting MS (RRMS) and primary progressive MS(PPMS). **(C)** Association between serum 25(OH) D levels between primary progressive MS group (PPMS) and second progressive MS (SPMS).

Serum 25(OH)D levels were significantly higher in patients with RRMS than in those with SPMS (WMD = 4.35, 95% CI: 2.16 to 6.54; **Figure 3A**). In contrast, no significant difference was found between RRMS and PPMS WMD= 4.56, 95% CI: −3. 18 to 12.31; **Figure 3B**), or between PPMS and SPMS (WMD = 0.30, 95% CI: −4.47to 5.06; **Figure 3C**).

### The aggregated WMD of the serum 25(OH) D in MS between summer and winter

**Figure 4:**
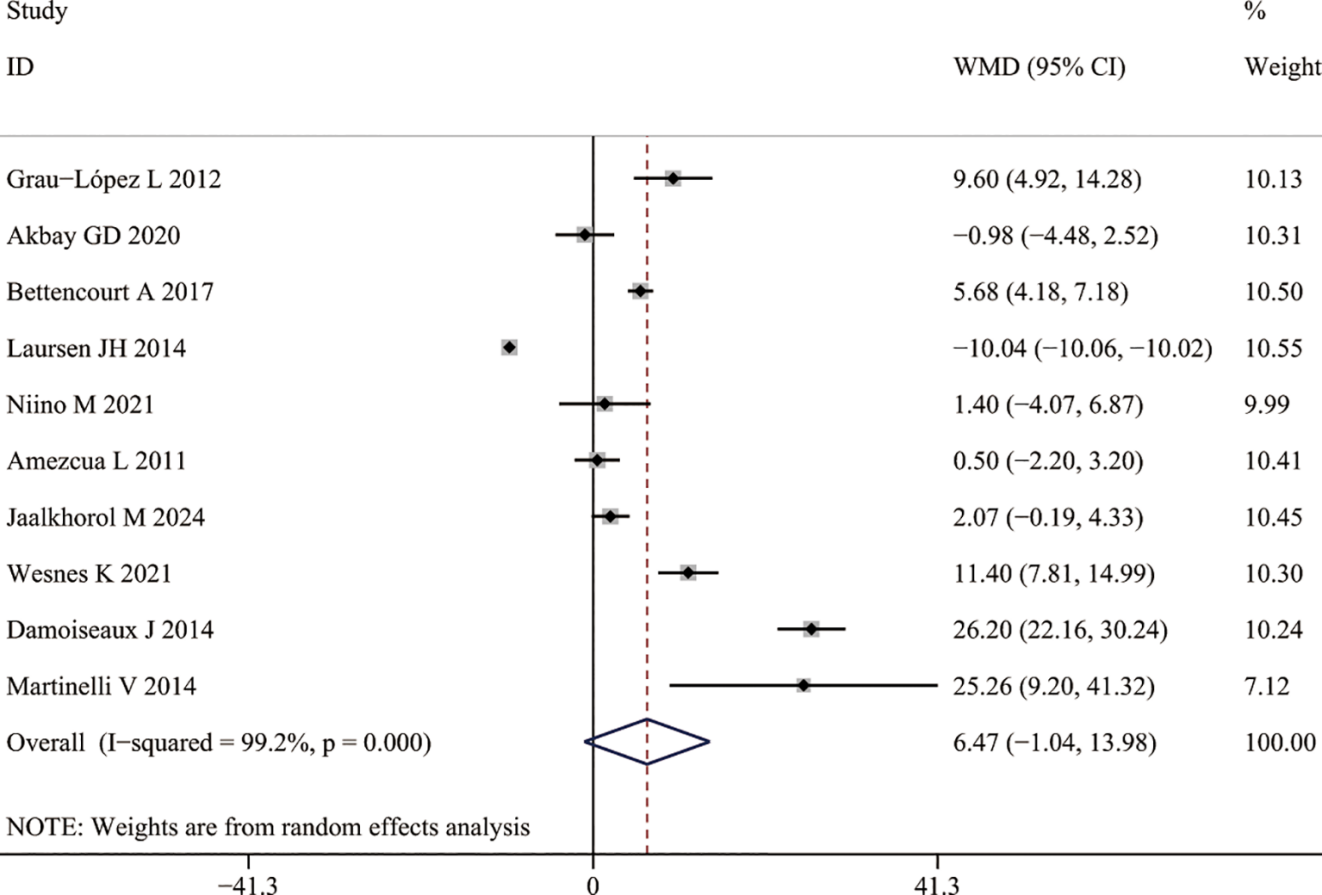
Serum 25(OH) D levels in MS between summer and winter.

No significant seasonal effect was observed for serum 25(OH) D levels, the aggregated WMD revealed no variation in serum 25(OH) D levels between winter and summer (WMD = 6.17[-1.04, 13.98]) (**Figure 4**).

### The multivariable-adjusted relative risk of MS, disease severity (EDSS) and disease activity for the highest compared with the lowest categories of serum 25(OH) D level

**Figure 5:**
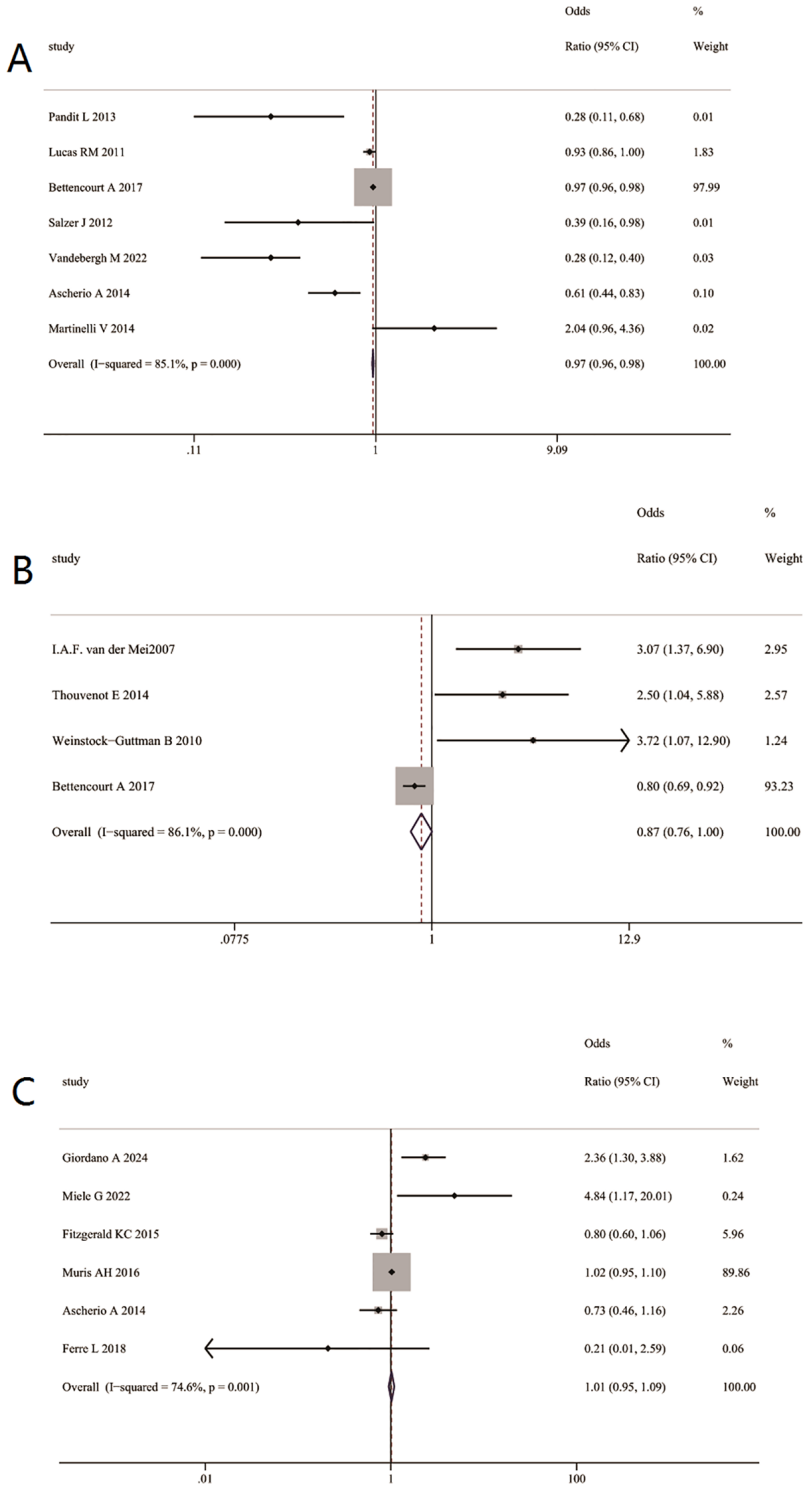
The multivariable-adjusted relative risk (RR) of MS outcomes for the highest versus lowest categories of serum 25(OH)D levels. **(A)** Association between serum 25(OH)D levels and risk of MS incidence. **(B)** Association between serum 25(OH)D levels and disease severity(EDSS). **(C)** Association between serum 25(OH)D levels and disease activity.

Multivariable-adjusted analysis comparing the highest versus lowest categories of serum 25(OH)D levels showed that higher 25(OH)D levels were associated with a lower risk of MS incidence (RR = 0.97, 95% CI 0.96–0.98; **Figure 5A**) and were inversely associated with disease severity as measured by EDSS (RR = 0.87, 95% CI 0.76–1.00; **Figure 5B**). In contrast, no significant association was observed between serum 25(OH)D levels and disease activity when comparing highest versus lowest categories (RR = 1.01, 95% CI 0.95–1.09; **Figure 5C**).

### Association between VDR polymorphisms and MS risk

**Figure 6:**
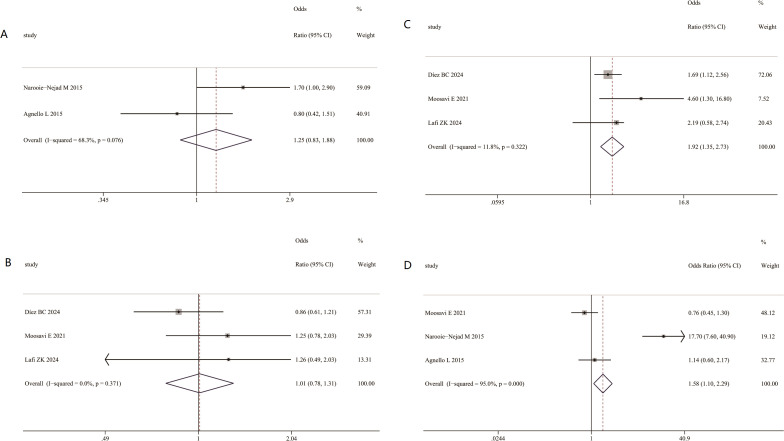
Association between VDR polymorphisms and MS risk. **(A)** Association between AC genotype for VDR ApaI(rs7975232) polymorphism and risk of MS **(B)** Association between CT genotype for VDR FokI (rs2228570) polymorphism and risk of MS **(C)** Association between TT genotype for VDR FokI (rs2228570) polymorphism and risk of MS. **(D)** Association between CT genotype for VDR TaqI(rs731236) polymorphism and risk of MS.

Our findings showed no association between AC genotype for VDR ApaI(rs7975232) polymorphism and risk of MS (OR = 1.25; 95% CI = 0.83– 1.88; **Figure 6A**); No association was found between CT genotype for VDR FokI (rs2228570) polymorphism and higher risk of MS (OR = 1.01; 95% CI = 0.78– 1.31; **Figure 6B**); We found that the TT genotype for VDR FokI (rs2228570) polymorphism was associated with higher risk of MS (OR = 1.92; 95% CI = 1.35–2.73; **Figure 6C**); CT genotype for VDR TaqI(rs731236) polymorphism was associated with higher risk of MS (OR = 1.58; 95% CI = 1.1–2.29; **Figure 6D**);

### Association of vitamin D3 supplementation with clinical outcomes in MS

**Figure 7:**
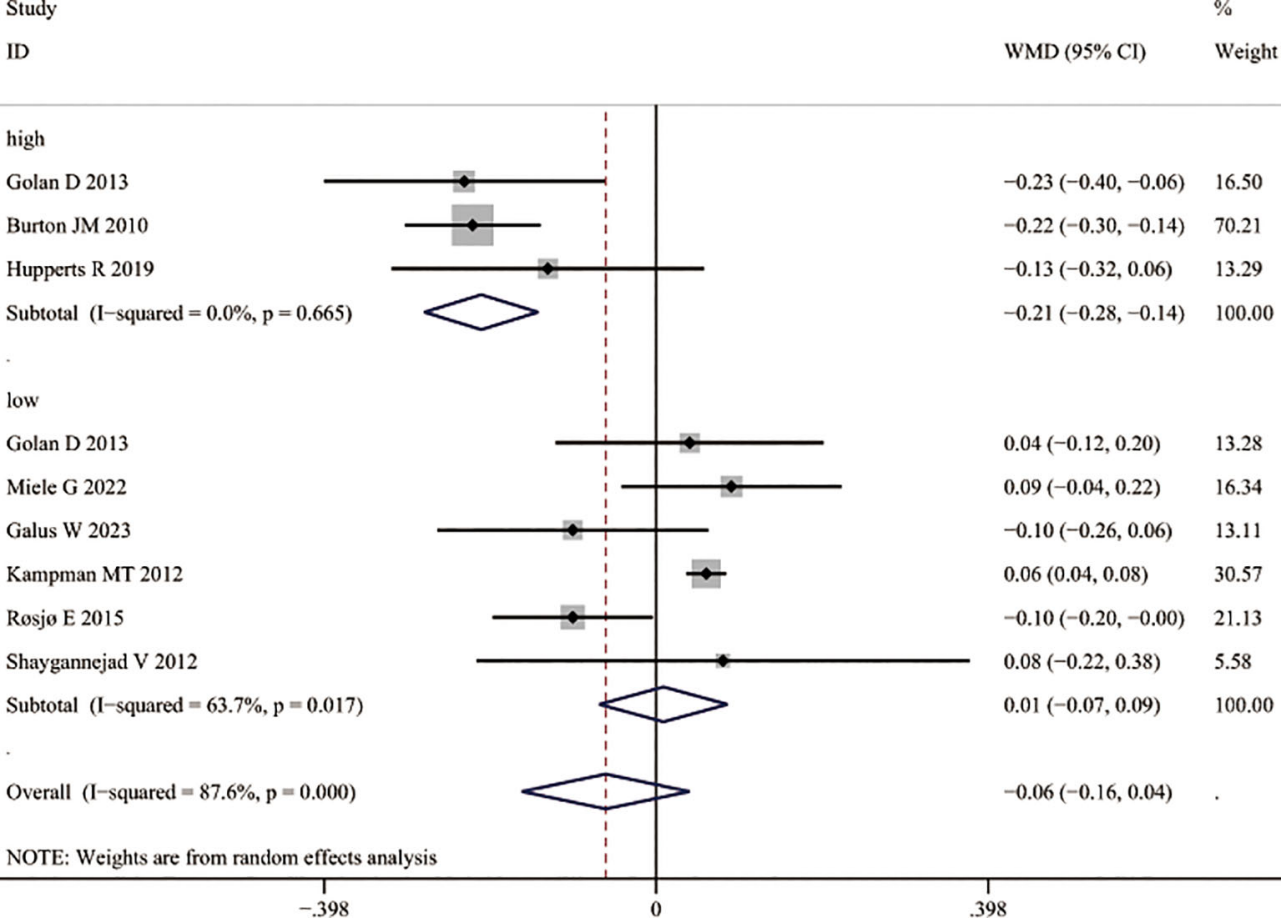
Association between vitamin D3 supplementation and annualized relapse rate (ARR) in MS.

Overall, vitamin D3 supplementation did not significantly reduce the ARR compared to placebo (WMD = –0.07, 95% CI –0. 18 to 0.03; **Figure 7**). However, when stratified by dose, high-dose supplementation was associated with a marked decrease in ARR (WMD = –0.21, 95% CI –0.28 to –0. 14; **Figure 7**), whereas no significant change was observed with low-dose supplementation (WMD = 0.01, 95% CI –0.07 to 0.09; **Figure 7**).

**Figure 8:**
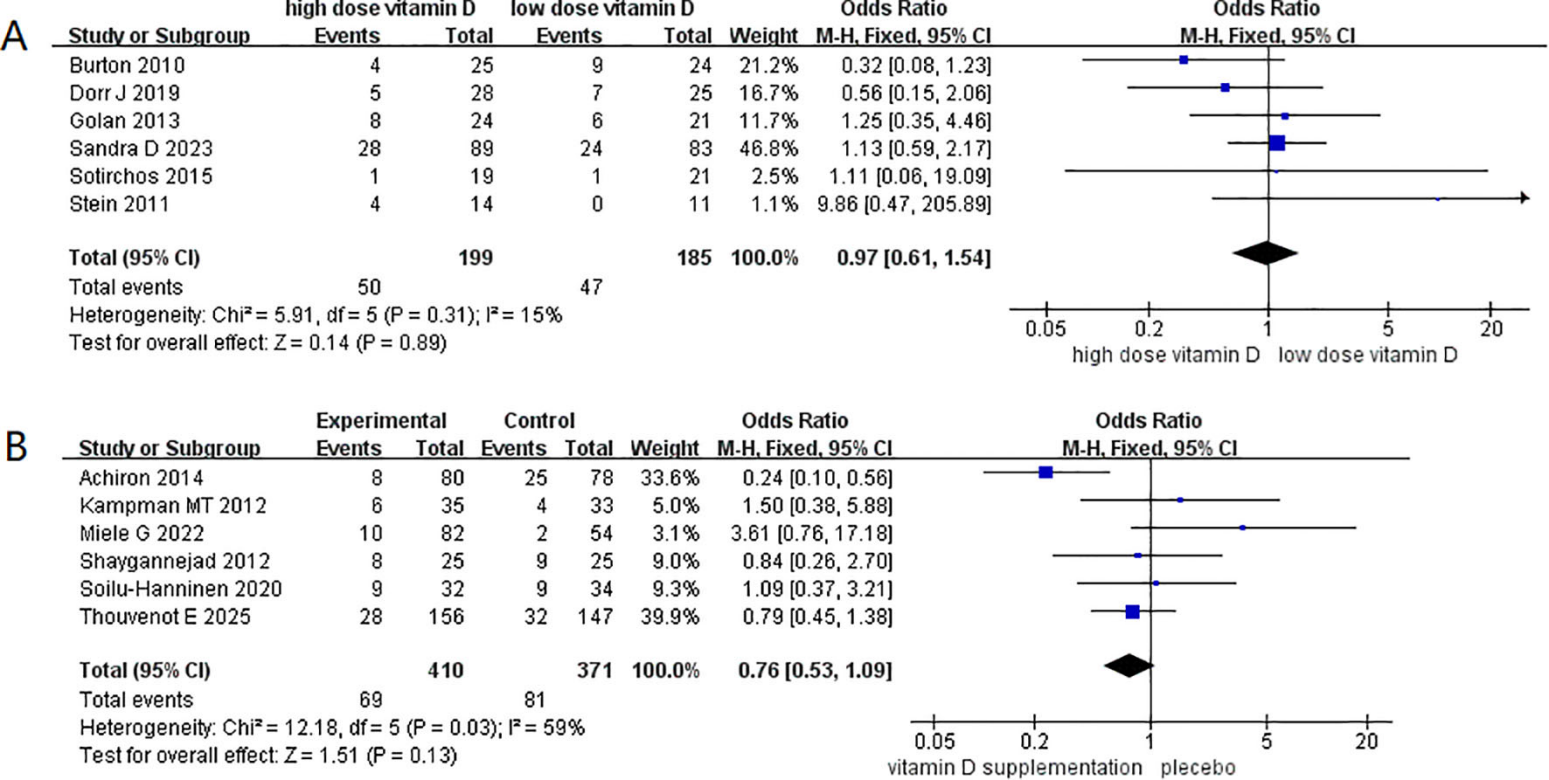
Relapse risk following vitamin D3 supplementation in patients with multiple sclerosis. **(A)** Relapse risk following high-dose and low-dose Vitamin D3 supplementation **(B)** Relapse risk following Vitamin D3 supplementation and placebo.

**Figure 9:**
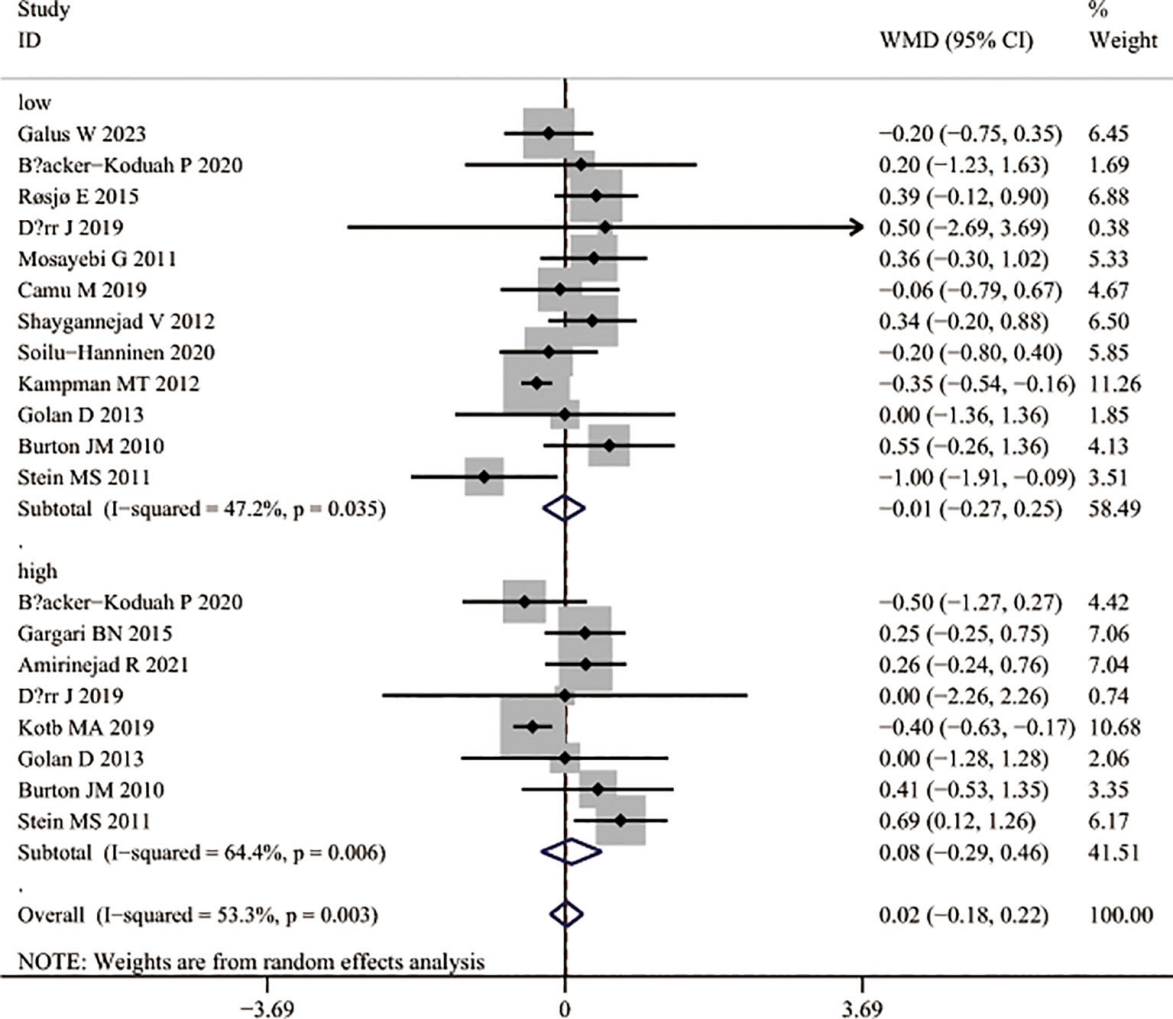
EDSS changes following vitamin D3 supplementation in patients with multiple sclerosis.

In analyses of relapse risk, no significant effect was found when comparing different dosage regimens (RR = 0.97, 95% CI 0.61– 1.54; **Figure 8A**) or supplementation versus control groups (RR = 0.76, 95% CI 0.53– 1.09; **Figure 8B**). Similarly, EDSS scores did not differ significantly between intervention and control groups after supplementation (WMD = 0.02, 95% CI –0. 18 to 0.22; **Figure 9**), and subgroup analysis showed no improvement in EDSS with either low-dose (WMD = 0.01, 95% CI –0.27 to 0.25) or high-dose (WMD = 0.08, 95% CI –0.29 to 0.46) regimens (**Figure 9**).

**Figure 10:**
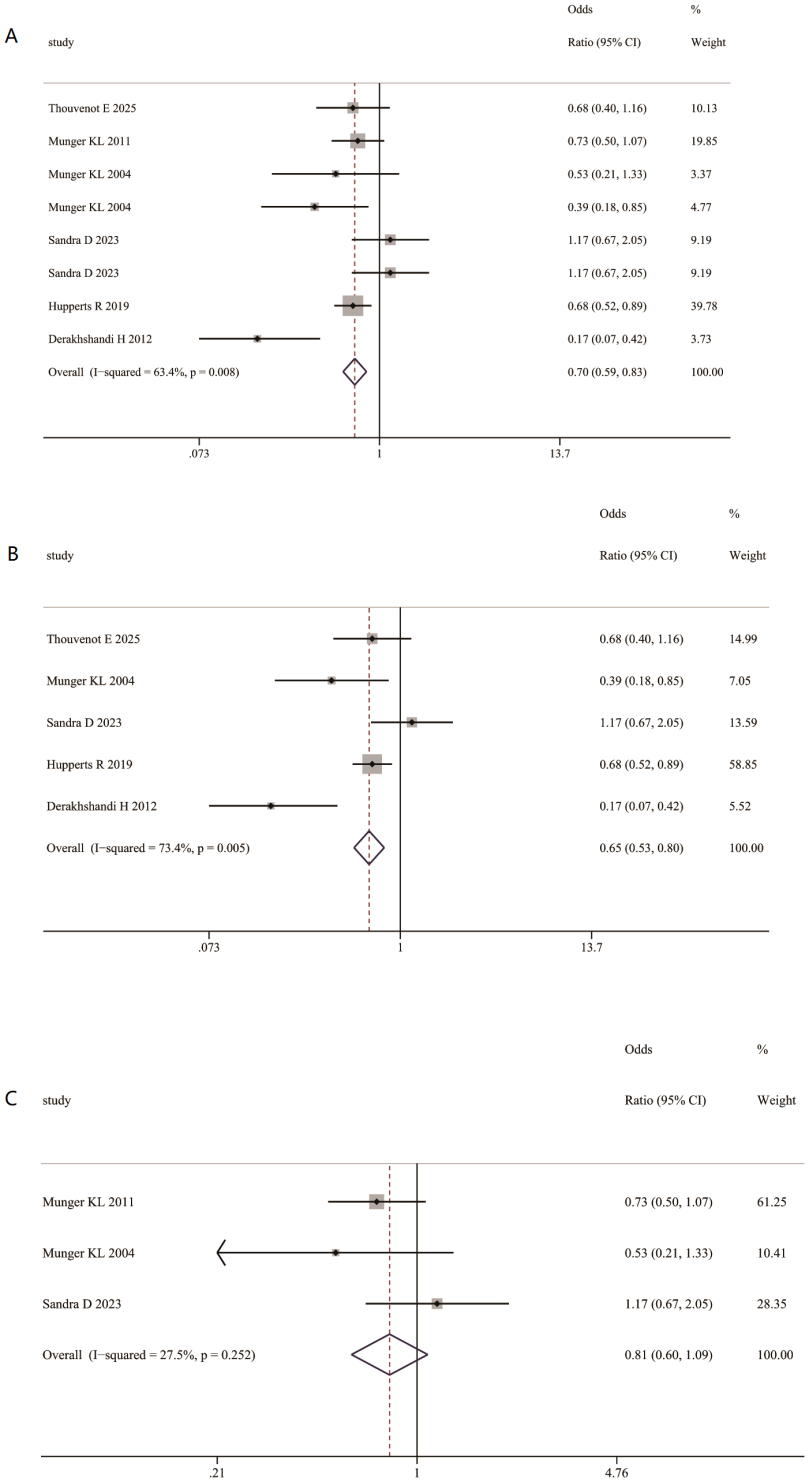
Association between Vitamin D3 supplementation and risk of MS relapse. **(A)** Vitamin D3 supplementation and risk of MS relapse. **(B)** High dose Vitamin D3 supplementation and risk of MS relapse. **(C)** Low dose Vitamin D3 supplementation and risk of MS relapse.

By contrast, multivariable- adjusted analysis indicated that vitamin D3 supplementation was associated with a reduced risk of MS relapse (RR = 0.70, 95% CI 0.59–0.83; **Figure 10A**). This protective association was significant only in the high-dose subgroup (RR = 0.65, 95% CI 0.53–0.80; **Figure 10B**), and not in the low-dose subgroup (RR = 0.81, 95% CI 0.60– 1.09; **Figure 10C**).

### Publication bias

**Figure 11:**
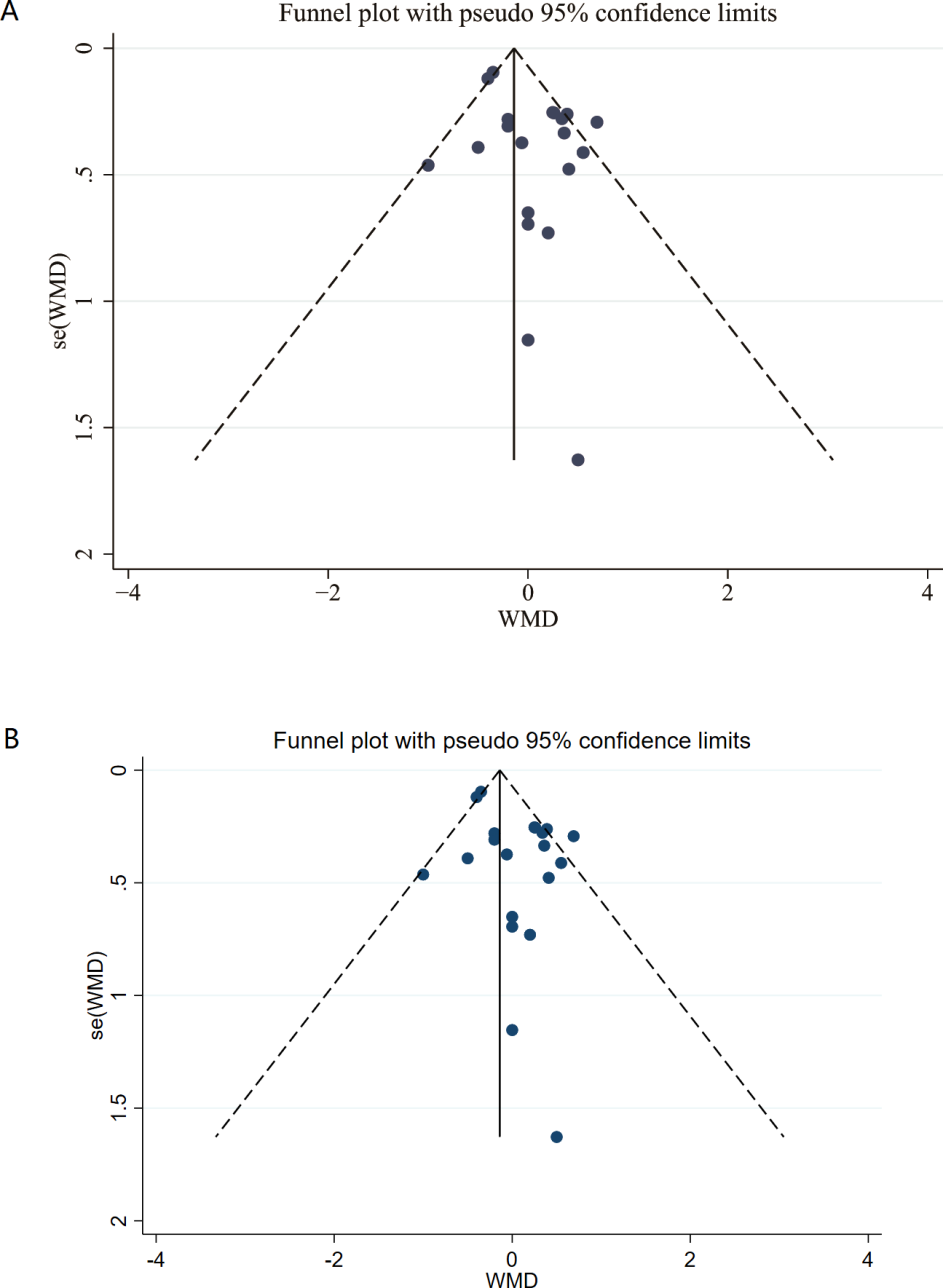
Funnel plot.

Visual inspection of the funnel plot revealed no evident asymmetry, indicating a low risk of publication bias. (**Figures 11A, B**).

## Discussion

This meta-analysis shows that serum 25(OH)D levels are significantly lower in patients with MS compared with healthy controls, with particularly marked reductions observed during relapse phases and in the secondary progressive MS subgroup. Serum 25(OH)D levels in MS patients did not show significant seasonal variation between summer and winter. When comparing the highest versus lowest categories of serum 25(OH)D levels, higher serum 25(OH)D concentrations were negatively correlated with both the incidence of MS and disease severity, as assessed by the EDSS, while these findings are consistent with a potential role of vitamin D in MS, the observational nature of the included studies precludes causal inference. VDR FokI polymorphism and VDR TaqI polymorphism were associated with higher risk of MS.Evidence indicates that high-dose vitamin D3 supplementation reduces the annualized relapse rate in MS, whereas low-dose supplementation does not produce a comparable effect. Overall, vitamin D3 supplementation at varying doses did not significantly influence relapse rates in MS patients, and no statistically significant difference was observed between the supplementation group and the control group. Furthermore, vitamin D3 intervention did not lead to improvements in EDSS scores.

Both animal and human studies have established 25(OH)D as a key immunomodulator, influencing regulatory T cells, B cells, and dendritic cells14-17. Given the central role of immune dysregulation in multiple sclerosis (MS), an inflammatory demyelinating disease of the central nervous system, the potential involvement of 25(OH) D in its pathogenesis has attracted substantial research interest (48–51). While numerous observational studies have examined the association between 25(OH) D levels and MS risk, their findings have been inconsistent (19, 52–54).

Several reports, including those by Khedr et al (52)., Correale et al (53)., and Akbay et al (54)., have demonstrated significantly lower serum 25(OH) D levels in MS patients compared with healthy controls, supporting a possible protective role of 25(OH)D. In contrast, other studies, such as those by Naiini et al (20)., have reported higher levels in patients, while work by Salzer et al (22)., Bhargawa et al (55)., and MoghtaderiA et al (56). found no statistically significant difference between groups. The present meta-analysis, which integrates available evidence, confirms that 25(OH)D levels are significantly reduced in individuals with MS relative to healthy controls, aligning with the former body of evidence (52) and suggesting that 25(OH)D deficiency may be implicated in MS susceptibility.

The clinical heterogeneity of MS has prompted in-depth investigation into differences in 25(OH)D levels across disease subtypes, disease phases, and seasonal variations. Although the influence of ultraviolet B(UVB) exposure and seasonal fluctuations on vitamin D metabolism is well-recognized (43), findings regarding their specific patterns in MS patients and their association with disease features remain inconsistent (28, 52, 53, 57). Our systematic review synthesizes and analyzes the relevant evidence. Studies suggest potential differences in 25(OH)D levels among MS subtypes. Some reports indicate significantly lower serum 25(OH)D levels in patients with SPMS compared to those with RRMS (52). Similarly, serum 25(OH)D levels in SPMS appear lower than in PPMS (28). The same study also found that RRMS patients with a longer disease duration (>5 years) and one or more relapses in the preceding two years had significantly lower serum 25(OH)D levels than RRMS patients without relapses during the same period (28). The findings of Thouvenot et al. further support higher serum 25(OH)D levels in RRMS patients compared to those with progressive forms of MS (including PPMS and SPMS), although no significant difference was observed between PPMS and SPMS (57). However, conflicting results exist; for example, Correale et al. reported lower serum 25(OH)D levels in RRMS patients compared to a PPMS control group (53). Our subgroup analysis revealed lower serum25(OH)D levels in SPMS than in RRMS, while no statistically significant differences were found between PPMS and either RRMS or SPMS.

Regarding the impact of disease activity (relapse vs. remission) in RRMS on 25(OH)D levels, study results are divergent. Candan et al. reported higher serum 25(OH)D levels during relapse compared to remission in RRMS patients (58). In contrast, Gö kçen et al. found that the mean 25(OH)D level in RRMS patients during remission was significantly lower than that in healthy controls (59). Similarly, Correale J et al. demonstrated lower serum 25(OH)D levels in RRMS patients, particularly during exacerbations, compared to healthy individuals, suggesting a potential link between serum 25(OH)D and MS disease activity (53). Our meta-analysis aligns with the latter studies, showing that the average serum 25(OH)D level during relapse was lower than that in the remission-phase in control group.

Seasonal fluctuation in 25(OH)D levels, typically higher in summer and lower in winter, is widely recognized and has been proposed to correlate with MS disease activity (17, 24, 60). Epidemiological and cross-sectional studies in Caucasian populations have confirmed this seasonal pattern (61). Among MS patients, significant seasonal differences have also been observed by Amezcua et al (62)., Damoiseaux et al (63)., and Niino et al (64). (in Japanese patients with mild to moderate disability). Nevertheless, other studies, such as that by Akbay et al (54)., found no significant variation in serum 25(OH)D levels between summer and winter in MS patients. While existing data generally indicate lower 25(OH)D levels in MS patients and suggest possible seasonal variation, robust statistical evidence supporting a consistent and definitive seasonal effect remains limited. Our comprehensive.

Additionally the potential influence of sex on vitamin D effects in MS warrants consideration. Epidemiological and clinical evidence indicates possible sex-based differences in vitamin D metabolism, circulating levels, and immunomodulatory responses, which may influence the relationship between 25(OH)D status and MS susceptibility or disease course. In the present meta-analysis, however, we could not conduct sex-specific subgroup analyses or adjust for potential effect modification by sex due to insufficient stratified data in the included studies. The limited reporting of sex-d isaggregated outcomes in the available literature represents an important gap. Future studies should be designed to systematically collect and analyze data by sex to clarify its role as a potential effect modifier.

Emerging evidence suggests an inverse relationship between circulating serum 25(OH)D levels and the risk of developing multiple sclerosis (MS) (15). Supporting this, Bettencourt et al. reported that lower serum 25(OH)D levels were associated with greater disease susceptibility and higher disability scores among MS patients in Portugal (20). The significance of vitamin D as a modifiable risk factor is further highlighted by findings of reduced serum 25(OH)D levels in individuals with recently diagnosed MS (15). Similarly, an independent cohort study in the United States indicated a decreased risk of MS among white adults with higher serum 25(OH)D concentrations (65). These observations align with earlier work by Souliu-Hä nninen et al., who also documented a correlation between serum 25(OH)D levels and relapse rate prior to assessment (61). Collectively, our systematic review corroborates an inverse association between serum 25(OH)D levels and MS incidence, indicating that lower serum 25(OH)D levels are linked to an increased probability of developing MS. These consistent findings across diverse populations underscore a likely important role for 25(OH)D in both the pathogenesis and clinical course of multiple sclerosis.

Our findings support an association between reduced serum 25(OH)D levels and the development of MS. The potential underlying mechanisms may involve the following pathways:1). Immunoregulatory Effects: Vitamin D exerts broad immunomodulatory actions by targeting key immune cell populations. It promotes immune tolerance by inhibiting the differentiation of pro-inflammatory T helper 17 (Th17) cells and their production of interleukin-17 (IL-17), while simultaneously enhancing the function and stability of regulatory T cells (Tregs). Furthermore, vitamin D modulates B-cell activity, reducing autoantibody production and inflammatory responses. It also influences monocytes and macrophages by suppressing the secretion of pro-inflammatory cytokines and promoting an anti-inflammatory phenotype (66, 67). 2) Direct Neuroprotection and Repair: Beyond systemic immunomodulation, vitamin D has direct protective and reparative roles within the central nervous system (CNS). It supports myelin repair by promoting the differentiation of oligodend rocyte precursor cells into mature, myelinating oligodend rocytes (28, 63). Vitamin D also contributes to the maintenance of blood-brain barrier (BBB) integrity, which is often compromised in early MS. It helps preserve BBB function by downregulating matrix metalloproteinases (MMPs) and upregulating the expression of tight junction proteins, thereby limiting the influx of harmful substances into the CNS (68) 3). Gene-Environment Interactions: The influence of vitamin D on MS risk involves complex interactions between genetic susceptibility and environmental factors. An individual’s genetic background can affect both vitamin D metabolism and its downstream immunologic effects. Notably, the promoter region of *HLA-DRB1* 1501-the strongest genetic risk variant for MS-contains a vitamin D response element(VDRE), suggesting that vitamin D levels may directly regulate its expression (69, 70).

Additionally, beyond circulating 25(OH) D levels, genetic variation in the VDR is a key factor potentially influencing individual susceptibility to MS and the biological response to vitamin D, highlighting how genetic variation may modify the biological impact of vitamin D status (71). The VDR gene harbors several polymorphisms (e.g., TaqI rs731236, ApaI rs7975232) whose associations with MS risk have been investigated with heterogeneous results (9–12). A notable study by Narooie-Nejad et al. in a Southeastern Iranian population reported an exceptionally strong association between the CC genotype and MS risk, with the TC genotype also conferring significant risk (9). Similarly, the ApaI CC genotype showed a positive association. Importantly, this study found no significant difference in serum 25(OH)D levels between patients and controls, suggesting the observed genetic associations may reflect alterations in VDR function or signaling rather than being secondary to vitamin D deficiency (9). Our findings in an Iranian population align partially with this, showing associations of FokI and TaqI, but not BsmI, polymorphisms with MS risk. These results are consistent with some reports in Japanese and American cohorts but contrast with studies from Turkey, Portugal, and the UK (10–12). Such discrepancies underscore the critical importance of population-specific genetic background in modifying disease risk. The reasons for this heterogeneity are likely multifaceted. Functionally, polymorphisms like FokI (rs2228570) and TaqI (rs731236) can alter VDR protein structure, stability, or transcriptional activity, thereby influencing vitamin D signaling efficiency (9–11). Furthermore, the genetic architecture governing vitamin D response extends beyond VDR. Variations in genes involved in vitamin D metabolism (e.g., CYP27B1, CYP24A1) and the major MS risk gene HLA-DRB1* 1501—whose promoter contains a vitamin D response element—may interact with VDR signaling, collectively affecting vitamin D bioavailability and immunomodulatory efficacy (72, 73). Supporting a direct genetic influence on vitamin D status, observational studies indicate that MS patients carrying the TaqI C/T genotype have significantly lower serum 25(OH)D levels, suggesting genetics can influence vitamin D status independently of supplementation. This may partly explain the highly variable clinical responses to standardizedvitamin D3 supplementation.

Sunlight is the primary source of vitamin D synthesis in humans. Variation in exposure— influenced by latitude, season, cultural clothing habits, and deliberate sun avoidance—can substantially affect circulating 25(OH)D levels independently of supplementation. Consequently, differences in sunlight exposure may introduce confounding in observational studies examining the association between 25(OH)D deficiency and the risk or progression of MS. During data extraction, we found that only two of the included studies adjusted for sunlight exposure as a confounder (23, 40). Those studies indicated an inverse association between higher serum 25(OH)D levels and MS, which remained significant after adjusting for sun exposure. Although geographic region was used as a rough proxy for ambient UV exposure, the lack of individual-level sunlight data represents a limitation of both the original studies and this meta-analysis. Future prospective studies should incorporate standardized measures of UV exposure, such as dosimetry or validated questionnaires, to better distinguish between the effects of endogenous synthesis and oral supplementation.

The relationship between serum25(OH) D levels and clinical severity in MS, primarily evaluated using the EDSS, has been widely studied with inconsistent conclusions. While higher levels of 25(OH)D have been associated with a reduced risk of MS and decreased disease activity— including lower relapse rates and slower disability progression—the overall association appears complex and subtype-dependent (74–77). Evidence from several studies supports an inverse correlation between serum 25(OH)D and disability measures (76, 78). For example,Smolders et al. reported that lower baseline 25(OH)D levels correlated with higher EDSS scores, a finding consistent with longitudinal observations linking serum 25(OH)D insufficiency to increased relapse risk (36, 53), greater MRI activity (79, 80), and accelerated disability accumulation (81). Similarly, van der Mei et al. observed that patients with higher disability (EDSS >3) had a greater prevalence of 25(OH) D deficiency (38), and Thouvenot et al.confirmed an inverse correlation between serum 25(OH)D levels and EDSS in a French MS cohort (57). Our meta-analysis aligns with these results, reinforcing an overall inverse relationship between circulating 25(OH)D and EDSS-assessed disease severity in MS. However, this correlation is not uniform across all studies or MS subtypes. Some research, particularly in relapsing-remitting MS (RRMS), has found no statistically significant association between serum 25(OH)D and EDSS scores (24). Furthermore, the strength of the inverse correlation appears more pronounced in progressive forms of MS (PPMS and SPMS) than in RRMS (43).

Mechanistically, the nature of this relationship remains unclear. Smolders et al. suggested that serum 1,25-dihydroxyvitamin D is not central to this association; instead, lower metabolite levels during relapses or in patients with higher EDSS may reflect intrinsic metabolic links or be secondary to the disease state (76). Two non-exclusive explanations are plausible: (1) chronically low serum 25(OH)D may directly exacerbate neuroinflammatory damage and disability progression (5), or (2) reduced mobility and sun exposure in patients with advanced disability may limit endogenous 25(OH) D synthesis, creating a reverse-causality loop (81). In summary, although low 25(OH)D levels are consistently observed in patients with greater disability or during relapses, the direction of causality remains ambiguous. Data from early RRMS patients suggest a temporal link between low 25(OH)D and relapses, supporting a potential modulatory role of 25(OH)D in disease activity (82, 83). While, its definitive therapeutic value in altering MS progression requires confirmation through rigorously designed,double-blind, placebo-controlled trials.

When evaluating the observed link between serum 25(OH)D levels and multiple sclerosis (MS), a key methodological issue in cross-sectional studies—reverse causation— must be clearly addressed. Individuals with MS, particularly those with advanced disability or progressive forms of the disease, often have reduced mobility, leading to less time outdoors and decreased sunlight exposure, the primary natural stimulus for cutaneous vitamin D production. Thus, the low 25(OH)D levels frequently seen in MS populations may reflect disease- related behavioral changes rather than a causative role in MS etiology. This inherent limitation substantially weakens causal inference from cross-sectional data, positioning such evidence primarily as hypothesis- generating. Furthermore, the causal relationship is strongly supported by Mendelian randomization (MR) studies. To date, major MR studies have consistently demonstrated that genetically determined lower lifelong levels of 25(OH)D are associated with a significantly increased risk of developing MS (84, 85). This robust genetic evidence indicates a potential causal, etiological role for vitamin D insufficiency in MS pathogenesis, independent of confounding environmental or behavioral factors. MR findings thus provide a crucial biological bridge between observational associations and the rationale for clinical intervention. In summary, while cross-sectional studies are limited by reverse causation, MR analyses strongly suggest a causal link between genetically lowered serum 25 (OH)D and MS risk. Nevertheless, the most clinically relevant evidence must come from large-scale, well-designed randomized controlled trials of vitamin D3 supplementation. Such RCTs are essential to determine whether correcting vitamin D deficiency can modify the disease course in established MS.

The association between serum 25(OH)D deficiency and MS has naturally prompted investigation into its therapeutic utility. However, clinical trial outcomes regarding the efficacy of vitamin D3 supplementation in altering disease course have been heterogeneous, likely due to variations in study design, population characteristics, dosing regimens, and formulation. Randomized controlled trials (RCTs) examining the impact of vitamin D3 on relapse rates have reported conflicting findings (86, 87). Some studies, including a 2025 trial by Thouvenot et al.’ suggest that high-dose supplementation (100,000 IU cholecalciferol biweekly) can reduce disease activity in early MS or clinically isolated syndrome (CIS) (88). Similarly, Burton et al. reported that very high daily doses(up to 40,000 IU) may decrease relapse rates (85), and Golan et al. observed a reduced ARR specifically in a high-dose group (89).

For instance, a large U.S. phase III trial found no difference in relapse rates between high- and low-dose vitamin D3 groups after 96 weeks (90). Kampman et al. also reported that weekly high-dose supplementation did not improve ARR (91),and Stein et al. demonstrated that high-dose vitamin D3 was no more effective than low-dose in reducing MRI lesions activity (92). The evidence for vitamin D3 supplementation slowing disability progression, as measured by the EDSS, is also inconsistent. While the CHOLINE RCT reported a statistically significant delay in EDSS progression with vitamin D3 compared to placebo94, other major trials such as SOLAR (93) and EVIDIMS (94) found no significant differences in EDSS scores between treatment and control groups or between different dosing arms. A prior meta-analysis by Hanaei et al., which excluded several key RCTs, concluded no significant effect on EDSS, highlighting the sensitivity of conclusions to the studies included (95).

Our systematic analysis of this body of evidence indicates that, overall, vitamin D3 supplementation does not consistently lead to statistically significant reductions in relapse rates or improvements in EDSS scores across all MS populations. However, subgroup analyses suggest a potential dose-dependent effect, with high-dose regimens showing more promise for reducing relapse risk in some studies, whereas low-dose supplementation consistently fails to demonstrate a significant benefit. The causal relationship remains difficult to disentangle, as the observed effects may be modified by disease stage, subtype, baseline 25(OH)D status, and genetic factors. Given these equivocal results, vitamin D3 supplementation cannot yet be recommended as a standard disease-modifying therapy for MS. Nevertheless, its relative safety profile supports its role as a nutritional adjunct.

Definitive conclusions regarding its therapeutic efficacy await further large-scale, long-term, and well-stratified randomized placebo-controlled trials.

It should be noted that in the included studies, some patients with multiple sclerosis (MS) were concurrently receiving various disease-modifying therapies (DMTs) during vitamin D3 supplementation or observational assessments. These treatments included, for example, interferon- β, glatiramer acetate, natalizumab, ocrelizumab, and fingolimod. While these agents primarily modulate immune activity in MS, they may potentially interact with vitamin D metabolism or serum levels. For instance, immunomodulatory therapies could influence inflammatory pathways that also intersect with vitamin D signaling. Some investigators have examined this relationship; notably, Kotb et al (96). reported that the increase in serum 25(OH)D levels following vitamin D3 supplementation was not affected by immunomodulatory treatment. However, due to heterogeneity in DMT regimens across studies and the lack of consistent reporting on interactions with vitamin D, our analysis was unable to adjust for this confounding factor. Future studies should consider stratifying patients by DMT status to clarify whether concomitant therapies modify the association between vitamin D3 and MS outcomes.

The heterogeneous results observed in clinical trials of vitamin D3 supplementation for MS underscore the complexity of its mechanism of action, which appears to be dose-dependent and context- specific.The following considerations may explain the divergence between biological plausibility and variable clinical efficacy.1) Dose-Dependent Immunomodulation: The immunomodulatory effects of vitamin D, considered its primary mechanism of action in MS, are highly dose-sensitive. High-dose regimens may suppress disease acticity by promoting immune tolerance, inhibiting pathogenic T-cell and B-cell responses, and downregulating pro-inflammatory cytokine production (15, 96). However, the activated immune system in MS presents a high threshold for regulation. Preclinical and clinical data suggest that achieving serum 25(OH)D concentrations above a certain therapeutic level is necessary to deliver sufficient signaling via the VDR pathway to meaningfully dampen inflammatory activity (97, 98). This pharmacodynamic requirement likely explains why significant reductions in relapse risk were observed only in the high-dose subgroup of our analysis, whereas low-dose supplementation proved ineffective.2). Challenges in Demonstrating Effects on ARR: The lack of a significant overall effect on ARR may be attributed to methodological and clinical factors inherent to MS trials. First, ARR is a continuous variable with inherent variability, requiring large sample sizes and prolonged follow-up to detect modest treatment effects, especially against the background of potent disease-modifying therapies (DMTs) (86). Second, in populations where patients are concurrently treated with high-efficacy DMTs that substantially lower baseline relapse rates, the potential additive benefit of vitamin D may be marginal and difficult to detect—a statistical ceiling effect (87). The heterogeneity in baseline ARR across included studies further complicates the pooled analysis.3). Limited Impact on EDSS: The absence of significant improvement in EDSS scores with vitamin D3 supplementation aligns with the scale’s reflection of accumulated, often irreversible, neuroaxonal damage (57, 99). While vitamin D may support neuroprotection, remyelination, and antioxidant pathways in experimental models, its capacity to reverse fixed neurological deficits in established MS is likely limited (49). The therapeutic window for influencing disability progression may be early in the disease course, possibly even preclinically, whereas most clinical trials enroll patients with existing diagnoses and varying degrees of permanent injury (5, 61). Furthermore,individual genetic differences in VDR sensitivity, polymorphisms, and vitamin D metabolism can create variability in tissue-level bioavailability and biological response, blunting the uniform clinical effect on a functional measure like the EDSS (86).

The safety profile of vitamin D3 supplementation, particularly at higher doses, represents an important consideration for clinical translation. Although vitamin D3 is generally well-tolerated, excessive intake may lead to hypercalcemia, hypercalciuria, and an increased risk of nephrolithiasis. Among the interventional studies included in this review, adverse events related to vitamin D3 supplementation were infrequently reported and mostly mild. Isolated cases of transient hypercalcemia or hypercalciuria were noted in a few trials (e.g., Sotirchos et al.; Hupperts et al.), yet no study reported serious renal complications or clinically significant stone events (29, 93). Importantly, several trials explicitly documented no increase in calcium-related adverse events or nephrolithiasis with high-dose regimens (77). These observations suggest that, under monitored conditions, high- dose vitamin D3 supplementation appears to have a favorable safety profile in patients with multiple sclerosis over the duration of the examined trials. However, the potential for long-term toxicity, especially with unsupervised, very high-dose supplementation, cannot be overlooked. Future trials should incorporate systematic monitoring of serum calcium, urinary calcium, renal function, and nephrolithiasis risk factors to better define the safety boundaries of vitamin D dosing in multiple sclerosis.

This meta-analysis primarily focused on the associations between serum 25(OH)D levels and MS incidence, relapse rate, and EDSS progression, as well as the effects of vitamin D3 supplementation on ARR and disability accrual. Although NEDA- 3 (no evidence of disease activity, defined as freedom from relapses, disability progression, and new MRI lesions) (100, 101) has emerged as a composite clinical endpoint in many disease- modifying therapy trials for MS, most of the interventional studies included in our review did not systematically report NEDA- 3 attainment— particularly at the 24- month timepoint. Consequently, we were unable to pool data on the impact of vitamin D3 supplementation on the proportion of patients achieving NEDA- 3. Future randomized controlled trials should more consistently adopt NEDA- 3 as a clinical endpoint, especially when evaluating the long- term disease- control effects of vitamin D3 supplementation. In summary, the therapeutic efficacy of vitamin D3 in MS is contingent upon achieving sufficient biological exposure, is most discernible in inflammatory outcomes like relapses, and may be attenuated in the context of advanced neurodegeneration or concurrent effective immunotherapies. These factors collectively explain the gap between promising mechanistic data and inconsistent clinical trial outcomes.

This meta-analysis is subject to several limitations that should be considered when interpreting its findings. First, the overall strength of the evidence is constrained by the predominance of observational case-control studies among the included literature, which are inherently more susceptible to confounding than randomized controlled designs. Second, while funnel plot symmetry suggested no major publication bias, the exclusion of non-English publications means this risk cannot be fully discounted. Meanwhile,this study is subject to certain selection bias. For instance, all included studies excluded pregnant women and patients under 15 years of age. Consequently, the findings of this study primarily apply to adult non-pregnant patients with multiple sclerosis and should not be directly extrapolated to the aforementioned populations.Third, the inability to perform subgroup analyses based on key demographic (e.g., age, sex, ethnicity) and clinical covariates (e.g., disease duration, concomitant therapies, lifestyle factors) limits the exploration of potential effect modifiers. Fourth, this meta-analysis included both observational and interventional studies to address complementary research questions. These study types were analyzed separately, and no combined synthesis of observational and interventional data was performed. The distinct methodological frameworks—observational studies for association and RCTs for efficacy—are reflected in the separate presentation of results and the cautious interpretation of findings throughout the Discussion. The inherent limitations of observational data, including potential confounding and reverse causation, are acknowledged, and causal claims are avoided. Heterogeneity in the design and outcome reporting among the included randomized trials may affect the precision of pooled estimates for interventional outcomes. These factors necessitate a cautious interpretation of the results. Finally, although our analysis compared 25(OH)D levels across RRMS, SPMS, and PPMS subtypes, we could not incorporate data on patients with Clinically Isolated Syndrome (CIS) due to the lack of a sufficient number of comparative studies meeting our inclusion criteria. This highlights the need for future research to specifically evaluate vitamin D status in CIS cohorts relative to established MS subtypes. In addition, several of the included studies were limited by small sample sizes, variable intervention durations, and high dropout rates, which may contribute to heterogeneity and affect the precision of the pooled estimates. Although random-effects models were applied to account for between-study variation, these limitations suggest that the results should be interpreted with caution, and further rigorously designed trials are needed to confirm our findings. Future studies should prioritize large-scale, long-term randomized controlled trials with low dropout rates, particularly focusing on the long-term efficacy and safety of high-dose vitamin D (e.g., ≥5,000 IU per day). Moreover, we could not conduct subgroup analyses by severity of vitamin D deficiency (e.g., severe vs. low) due to inconsistent definitions and cut- offs across studies, preventing unified classification. This may obscure a dose– response relationship between vitamin D status and MS outcomes. Future studies should use standardized deficiency criteria to allow more detailed stratification. Additionally, although we extracted available safety data, heterogeneity in adverse event reporting across trials precluded a formal meta-analysis of side effects,particularly for rare but serious outcomes such as nephrolithiasis.

## Conclusions

This meta-analysis shows that serum 25(OH)D levels are significantly lower in patients with MS compared to healthy controls. An inverse association was found between 25(OH)D levels and both MS risk and disease severity. These findings highlight an association between 25(OH)D status and MS, but given the observational nature of the included studies, causality cannot be established. VDR gene polymorphisms were associated with MS risk. While high-dose vitamin D3 supplementation was associated with a reduction in relapse rates in some analyses, overall, supplementation did not yield statistically significant benefits on relapse rates or EDSS scores compared to control groups.

To clarify the therapeutic potential of 25(OH)D in MS, future research should prioritize well-designed, large-scale randomized controlled trials that address several key gaps: 1) Optimal Dosing: Establishing dose-response relationships and defining therapeutic ranges for specific clinical goals, such as relapse prevention versus slowing disability progression. 2) Integrated Biomarker Assessment: Correlating changes in serum 25(OH)D levels with relevant immunological, radiographic, and neuroprotective biomarkers to better understand the mechanism of action. 3) Patient Stratification: Investigating whether clinical efficacy varies across MS phenotypes, disability stages, genetic backgrounds, or in the context of different disease-modifying therapies. The precise role and optimal regimen for vitamin D3 supplementation in MS management remain to be determined. Definitive trials are needed to establish whether it can serve as a safe and effective adjunctive therapy for improving long-term outcomes in this patient population.

## Data Availability

The datasets presented in this study can be found in online repositories. The names of the repository/repositories and accession number(s) can be found below: guoshigang2025@163.com.
